# Association of Pre-Spinal Shock Indices and Right Internal Jugular Vein Collapsibility Index with Post-Spinal Hypotension During Cesarean Delivery: A Prospective Observational Study

**DOI:** 10.3390/jcm15155842

**Published:** 2026-07-26

**Authors:** Oğuz Özakın, Serpil Şehirlioğlu, Fatma Çiğdem Özakın, Döndü Genç Moralar, Veysel Dinç

**Affiliations:** Department of Anesthesiology and Reanimation, Health Sciences University Gaziosmanpaşa Training and Research Hospital, Istanbul 34255, Turkey; drserpilsahin@gmail.com (S.Ş.); f.cigdemulus@gmail.com (F.Ç.Ö.); dondugencm@gmail.com (D.G.M.); drvdinc@gmail.com (V.D.)

**Keywords:** cesarean delivery, diastolic shock index, internal jugular vein, modified shock index, postspinal hypotension, shock index, spinal anesthesia

## Abstract

**Background and Objectives**: Postspinal hypotension (PSH) remains a common complication of cesarean delivery under spinal anesthesia. Simple prespinal predictors may assist in identifying patients at increased risk. This study evaluated the associations of shock index (SI), modified shock index (MSI), diastolic shock index (DSI), and right internal jugular vein collapsibility index (RJV-CI) with postspinal hypotension (PSH). **Materials and Methods**: This prospective observational study included term parturients undergoing elective cesarean delivery under spinal anesthesia. Prespinal SI, MSI, DSI, and RJV-CI were measured before intrathecal injection. PSH was defined as systolic arterial pressure <90 mmHg or a reduction of ≥30% from baseline within 15 min after intrathecal injection. Receiver operating characteristic (ROC) analyses were performed for individual predictors. Three logistic regression models combining RJV-CI with SI, MSI, or DSI were evaluated using bootstrap internal validation. **Results**: Of 80 enrolled patients, 76 were included in the final analysis; 36 (47.4%) developed PSH. SI, MSI, DSI, and RJV-CI were significantly higher in the PSH group than in the non-PSH group. The areas under the ROC curve (AUCs) were 0.757 for SI, 0.747 for MSI, 0.693 for DSI, and 0.709 for RJV-CI. In multivariable models, RJV-CI remained significantly associated with PSH after adjustment for the respective shock index. The model combining RJV-CI and SI showed the highest apparent AUC (0.790; 95% confidence interval, 0.688–0.886) and an optimism-corrected AUC of 0.775. The RJV-CI plus MSI model showed an optimism-corrected AUC of 0.774. **Conclusions**: Prespinal SI, MSI, DSI, and RJV-CI were associated with PSH in this cohort. Models combining RJV-CI with SI or MSI showed numerically higher discrimination than the individual predictors. The derived cutoff values should be considered exploratory and require external validation before routine clinical use.

## 1. Introduction

Postspinal hypotension (PSH) is one of the most common and clinically important complications during cesarean delivery performed under spinal anesthesia [1]. The incidence of PSH varies across studies and has been reported to range from 39% to 75%; it may cause maternal symptoms such as nausea, vomiting, and dizziness and may adversely affect fetal oxygenation and acid–base status [2].

Given the rapid onset and potentially unpredictable nature of hypotension after spinal anesthesia, several preventive strategies have been proposed, including preoperative volume loading, vasopressor prophylaxis, and patient positioning. However, these measures are not universally effective and are often not individualized. Current preventive strategies are generally applied according to institutional practice and clinical judgment rather than based on an individualized, validated prespinal risk assessment. A simple bedside approach that identifies patients at increased risk before intrathecal injection could therefore help facilitate closer surveillance and support future risk-stratified preventive strategies [3].

Consequently, interest has shifted toward simple, noninvasive, and reliable predictors that may enable early risk stratification before spinal anesthesia [1,3]. One such parameter is the shock index (SI), defined as the ratio of heart rate (HR) to systolic arterial pressure (SAP) [4]. Although initially proposed for trauma and hemorrhagic shock, SI has also been investigated as a predictor of hemodynamic instability in sepsis, postpartum hemorrhage, and emergency intubation [4,5,6]. In obstetric populations, a prespinal SI ≥ 0.9 has been associated with a higher incidence of intraoperative and postdelivery hypotension, although its discriminatory performance when used alone appears limited [2].

The internal jugular vein collapsibility index (IJV-CI), measured using bedside ultrasonography, has also been evaluated as a noninvasive marker of intravascular volume status [7,8]. Compared with the inferior vena cava (IVC), the internal jugular vein (IJV) offers a particularly practical sonographic window in pregnant women. Its superficial cervical location allows direct visualization with a high-frequency linear probe, requires minimal patient repositioning, and does not depend on a subcostal abdominal acoustic window. These features may be advantageous in late pregnancy, when transabdominal IVC imaging can be technically challenging because of pregnancy-related anatomical changes and maternal body habitus [7]. IJV collapsibility index has been associated with fluid responsiveness and hypotension risk in both obstetric and nonobstetric populations [7,9,10,11]. Some recent studies have reported higher IJV-CI values in patients who developed PSH, suggesting a potential role in preoperative risk assessment [7,12]. Nevertheless, the relative predictive value of shock indices and ultrasonographic venous collapsibility for PSH remains insufficiently defined [1].

Most previous studies have evaluated these parameters separately or in relatively small cohorts. Prospective data assessing hemodynamic indices and venous collapsibility together in the same clinical setting remain limited. In addition, clinically applicable threshold values have not been consistently established, particularly in obstetric populations. Previous findings regarding shock indices and jugular venous collapsibility have also varied across study settings [1,7,12]. Therefore, whether these readily available measures provide complementary information when assessed together in women undergoing elective cesarean delivery remains uncertain.

Accordingly, the present study aimed to evaluate the associations of SI, modified shock index (MSI), diastolic shock index (DSI), and right internal jugular vein collapsibility index (RJV-CI) with postspinal hypotension in patients undergoing cesarean delivery under spinal anesthesia. We also explored whether combining RJV-CI with hemodynamic indices was associated with improved model discrimination for preoperative risk stratification. This analysis was intended as an exploratory assessment of association and discrimination rather than as the development of a definitive clinical prediction model.

## 2. Materials and Methods

### 2.1. Study Design and Ethical Approval

This prospective observational study was conducted at Health Sciences University Gaziosmanpaşa Training and Research Hospital between June 2023 and October 2023. The protocol was approved by the local Institutional Ethics Committee (Decision No: 2023/85), and the study was conducted in accordance with the Declaration of Helsinki. Written informed consent was obtained from all participants. The study was registered at ClinicalTrials.gov on 12 July 2023 (NCT05953129). This study was reported in accordance with the STROBE statement.

### 2.2. Patient Selection

Eligible participants were term parturients aged 18–45 years who were consecutively recruited and scheduled for elective cesarean delivery under spinal anesthesia, had an American Society of Anesthesiologists (ASA) physical status of II-III, and had no contraindications to spinal anesthesia. Exclusion criteria included severe cardiac disease, hypertensive disorders of pregnancy (preeclampsia, eclampsia, or HELLP syndrome), coagulopathy, known allergy to local anesthetics, refusal to provide consent, and conversion to general anesthesia. A total of 80 patients were enrolled; four required conversion to general anesthesia and were excluded, leaving 76 patients for analysis.

### 2.3. Anesthesia Technique and Monitoring

Standard monitoring included noninvasive blood pressure (NIBP), electrocardiography, and pulse oximetry. All patients underwent elective cesarean delivery according to the institution’s routine obstetric anesthesia practice. Baseline prespinal measurements of heart rate (HR), systolic arterial pressure (SAP), diastolic arterial pressure (DAP), and mean arterial pressure (MAP) were obtained three times at 1-min intervals, and the mean values were used for analysis.

The volume of intravenous fluid administered in the delivery ward before transfer to the operating room was recorded for each patient. No fixed-volume prophylactic fluid preload or prophylactic vasopressor was administered before spinal anesthesia to minimize alteration of prespinal hemodynamic measurements.

Spinal anesthesia was performed with patients in the seated position at the L3–L4 or L4–L5 interspace using a 25-gauge Quincke needle. After confirmation of free cerebrospinal fluid flow, 12 mg of 0.5% hyperbaric bupivacaine was injected at a controlled rate. Patients were then immediately returned to the supine position with left uterine displacement maintained. This was achieved by tilting the operating table. Sensory block level was assessed using the pinprick test after intrathecal injection, and surgery was commenced after a sensory block level of T4 had been confirmed.

NIBP and HR were recorded immediately after intrathecal injection, at 3-min intervals during the first 15 min, and thereafter at clinically appropriate intervals. Postspinal hypotension (PSH) was defined as SAP < 90 mmHg or a reduction in SAP of ≥30% from baseline within the first 15 min after intrathecal injection. At the onset of hypotension, intravenous ephedrine was administered in 5–10 mg doses and repeated as needed to restore SAP to within 20% of the baseline value. Rapid crystalloid infusion was administered concurrently during hypotensive episodes. Bradycardia, defined as HR < 50 beats/min, was treated with 0.5 mg of intravenous atropine.

### 2.4. Ultrasound Protocol: Right Internal Jugular Vein Collapsibility Index

Prespinal ultrasound assessment was performed using a standardized protocol to ensure consistency of measurement. The assessment was performed after routine monitoring had been applied in the operating room and before spinal anesthesia was administered. Patients were positioned supine with a 15° head-up tilt to standardize the hydrostatic reference point and a 15° left lateral tilt to minimize aortocaval compression.

Ultrasound assessments were performed using a MyLabSeven ultrasound system (Esaote, Genoa, Italy) with a high-frequency linear probe (6–13 MHz). The probe was placed transversely approximately 2 cm superior to the right sternoclavicular joint. Minimal probe pressure was applied to preserve native venous geometry. The probe was held perpendicular to the skin, and care was taken to avoid external compression of the vein during image acquisition. All ultrasound measurements were completed before intrathecal injection during quiet spontaneous breathing.

The right internal jugular vein (RJV) was continuously observed for three consecutive respiratory cycles using B-mode imaging. The maximum anteroposterior diameter (RJVmax) and minimum anteroposterior diameter (RJVmin) were recorded during quiet spontaneous respiration. Patients were instructed not to speak or perform a Valsalva maneuver during measurement. The anteroposterior diameter of the ipsilateral common carotid artery (CA) was measured in the same transverse view at end-expiration. The mean RJV diameter (RJVmean) was calculated as the mean of RJVmax and RJVmin. Jugular-to-carotid ratios, including RJVmax/CA, RJVmin/CA, and RJVmean/CA, were calculated.

To reduce measurement variability, two trained investigators independently obtained two measurements each, and the arithmetic mean of the four measurements was used for analysis. The investigators performing ultrasound did not participate in intraoperative blood pressure management and completed the ultrasound assessment before spinal anesthesia and outcome assessment. The anesthesiologist responsible for spinal anesthesia and hypotension management was blinded to the prespinal ultrasound measurements and shock index values to reduce observer bias.

The right internal jugular vein collapsibility index (RJV-CI) was calculated using the following formula:

RJV-CI = [(RJVmax − RJVmin)/RJVmax] × 100

### 2.5. Hemodynamic Indices

Prespinal hemodynamic indices were calculated from baseline values as follows: shock index (SI) = HR/SAP; modified shock index (MSI) = HR/MAP; and diastolic shock index (DSI) = HR/DAP.

### 2.6. Outcomes and Grouping

The primary outcome was PSH occurring within the first 15 min after intrathecal injection, as defined above. Participants were classified into PSH and non-PSH groups according to the presence or absence of the primary outcome. All measurements were obtained before intrathecal injection, and outcome assessment was restricted to the predefined 15-min interval after intrathecal injection.

### 2.7. Sample Size

Before initiating the main study, a pilot phase including 10 consecutive patients who underwent cesarean delivery under spinal anesthesia was conducted to assess feasibility and estimate the incidence of PSH in our institutional setting. In this pilot cohort, PSH occurred in 4 of 10 patients (40%), which informed the anticipated event rate for subsequent sample size planning.

For multivariable modeling, sample size was guided by an events-per-variable (EPV) approach. Assuming two predictors per model and a conservative EPV of 15, at least 30 hypotension events were required. Based on the pilot-estimated PSH incidence of 40%, the target sample size for the main study was 75 participants. To account for potential exclusions and support model stability, 80 patients were enrolled.

### 2.8. Statistical Analysis

Statistical analyses were performed using Python version 3.10 (Python Software Foundation, Wilmington, DE, USA) with the scikit-learn, statsmodels, and pandas libraries. A two-sided *p* value < 0.05 was considered statistically significant. No missing values were present for the variables included in the analyses. Continuous variables were assessed for normality using the Shapiro–Wilk test. Normally distributed continuous variables are presented as mean ± standard deviation (SD), whereas non-normally distributed variables are presented as median (minimum–maximum). Categorical variables are presented as numbers and percentages.

Comparisons between the PSH and non-PSH groups were performed using the independent-samples *t*-test for normally distributed continuous variables and the Mann–Whitney U test for non-normally distributed continuous variables. Categorical variables were compared using the chi-square test or Fisher’s exact test, as appropriate.

Receiver operating characteristic (ROC) curve analysis was performed to evaluate the discriminatory performance of SI, MSI, DSI, and RJV-CI for PSH. The area under the ROC curve (AUC), sensitivity, and specificity were calculated, and optimal cutoff values were determined using Youden’s index. The 95% confidence intervals for the AUC values were estimated using 1000 bootstrap resamples.

To evaluate the performance of combined models, three multivariable logistic regression models were fitted: Model A (RJV-CI + SI), Model B (RJV-CI + MSI), and Model C (RJV-CI + DSI). Odds ratios (ORs) with 95% confidence intervals (CIs) were calculated. ORs for RJV-CI were reported per 1% increase, whereas ORs for SI, MSI, and DSI were reported per 0.1-unit increase to facilitate clinical interpretation.

Model discrimination was quantified using the AUC. Internal validation was performed using 1000 bootstrap resamples to estimate optimism-corrected AUC values. Model calibration was assessed using the Hosmer–Lemeshow goodness-of-fit test; a *p* value > 0.05 was interpreted as no evidence of lack of fit. An exploratory decision-curve analysis was performed to evaluate the net benefit of the individual and combined models across a range of threshold probabilities (Supplementary Figure S1). Exploratory apparent calibration plots were generated for the combined models to visually assess agreement between predicted and observed probabilities (Supplementary Figure S2).

## 3. Results

**Figure 1:**
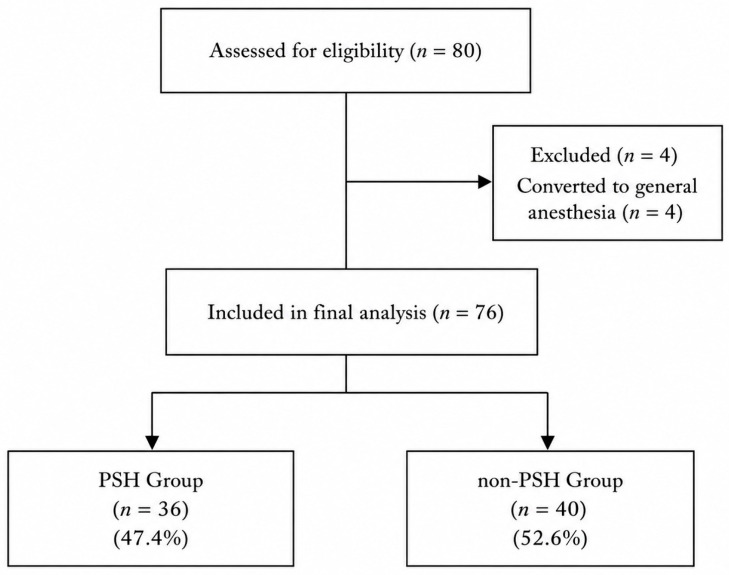
Flow diagram of the study.

Eighty patients were enrolled; four were excluded because of conversion to general anesthesia, leaving 76 patients for the final analysis (Figure 1). Of the 76 patients, 36 (47.4%) developed PSH. Demographic and baseline clinical characteristics were comparable between the groups, with no significant differences in age, anthropometric parameters, ASA physical status, fasting duration, preoperative fluid administration volume, or hematologic indices (all *p* > 0.05; Table 1).

Screening, exclusion due to conversion to general anesthesia (*n* = 4), and the final analysis groups are shown: PSH group (*n* = 36) and non-PSH group (*n* = 40).

### 3.1. Prespinal Hemodynamic Indices and Jugular Ultrasound

Patients who developed PSH had significantly higher prespinal shock-derived indices than those who did not develop PSH (Table 2). Mean SI was 0.84 ± 0.14 in the PSH group and 0.71 ± 0.12 in the non-PSH group (*p* < 0.001). Mean MSI was 1.16 ± 0.21 and 0.99 ± 0.18, respectively (*p* < 0.001), whereas mean DSI was 1.52 ± 0.28 and 1.32 ± 0.27, respectively (*p* = 0.004).

The prespinal RJV-CI was also higher in the PSH group than in the non-PSH group (44.22 ± 18.54% vs. 31.18 ± 15.74%, *p* = 0.002). The RJVmax/CA, RJVmin/CA, and RJVmean/CA ratios did not differ significantly between groups (*p* = 0.657, *p* = 0.091, and *p* = 0.520, respectively).

### 3.2. Univariable Diagnostic Performance

**Figure 2:**
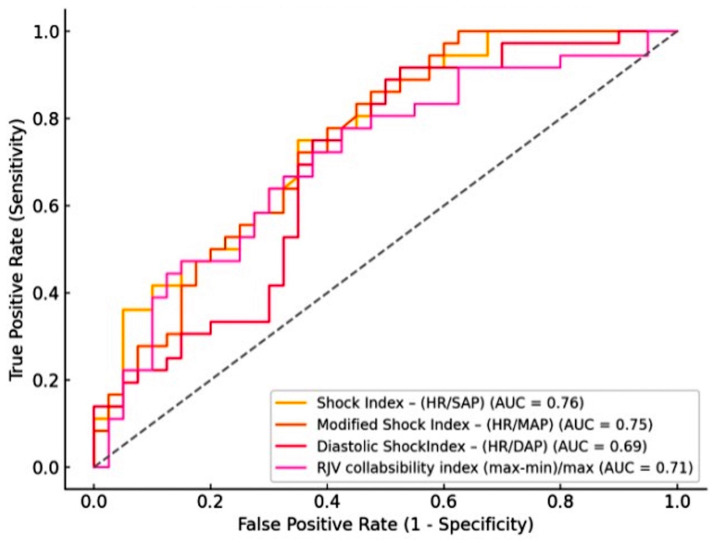
Receiver operating characteristic (ROC) curves for prespinal predictors of postspinal hypotension. The dashed diagonal line represents the no-discrimination reference line (AUC = 0.5).

The results of ROC analyses for the individual predictors are summarized in Table 3. Figure 2 presents the ROC curves for SI, MSI, DSI, and RJV-CI.

SI and MSI showed similar discriminatory performance, with AUCs of 0.757 and 0.747, respectively. SI had a sensitivity of 75.0% and specificity of 65.0%. MSI showed higher sensitivity (86.1%) but lower specificity (52.5%). DSI had an AUC of 0.693 and the highest sensitivity (91.7%), with a specificity of 47.5%. RJV-CI had an AUC of 0.709, with a sensitivity of 77.8% and specificity of 57.5%.

Receiver operating characteristic curves for SI, MSI, DSI, and RJV-CI. The AUCs, optimal cutoff values, sensitivities, and specificities were as follows: SI, AUC 0.757, cutoff 0.742, sensitivity 0.750, specificity 0.650; MSI, AUC 0.747, cutoff 0.976, sensitivity 0.861, specificity 0.525; DSI, AUC 0.693, cutoff 1.254, sensitivity 0.917, specificity 0.475; and RJV-CI, AUC 0.709, cutoff 30.488%, sensitivity 0.778, specificity 0.575.

### 3.3. Multivariable Prediction Models

Three multivariable logistic regression models combining RJV-CI with individual hemodynamic indices were evaluated (Table 4). In each model, RJV-CI remained significantly associated with PSH after adjustment for the respective shock index (all *p* < 0.05).

Model A, including RJV-CI and SI, showed the highest apparent discrimination, with an AUC of 0.790 (95% CI, 0.688–0.886). Following bootstrap internal validation, the optimism-corrected AUC was 0.775. The Hosmer–Lemeshow test showed no evidence of lack of fit (*p* = 0.559).

Model B, including RJV-CI and MSI, had an apparent AUC of 0.787 and an optimism-corrected AUC of 0.774. The Hosmer–Lemeshow test similarly showed no evidence of lack of fit (*p* = 0.597).

Model C, including RJV-CI and DSI, had an apparent AUC of 0.763 and an optimism-corrected AUC of 0.748. The Hosmer–Lemeshow test showed no evidence of lack of fit (*p* = 0.398).

Overall, the models combining RJV-CI with SI or MSI showed numerically higher discrimination than their respective individual predictors in this cohort. These findings suggest that RJV-CI may provide complementary information when interpreted alongside prespinal shock indices; however, given the modest differences in discrimination and the absence of formal incremental-value analyses, the observed model performance and proposed cutoff values should be considered exploratory and require validation in independent cohorts. In exploratory decision-curve analysis, the models combining RJV-CI with SI or MSI showed greater net benefit than the corresponding individual predictors across selected intermediate threshold-probability ranges. RJV-CI alone provided more limited net benefit. Because this analysis was performed in the development cohort and the curves were variable at higher thresholds, the findings should be interpreted cautiously (Supplementary Figure S1). Exploratory apparent calibration plots visually comparing predicted and observed PSH probabilities for Models A, B, and C are presented in Supplementary Figure S2.

## 4. Discussion

In this prospective study of patients undergoing cesarean delivery, prespinal bedside measures, including SI, MSI, DSI, and RJV-CI, were associated with the development of PSH. The models combining RJV-CI with SI or MSI showed numerically higher discrimination than the corresponding individual predictors, and the Hosmer–Lemeshow tests showed no evidence of lack of fit. These findings support further evaluation of simple, noninvasive measures for preoperative risk assessment before spinal anesthesia.

Spinal anesthesia-induced sympathectomy reduces venous return and systemic vascular resistance in parturients who are already susceptible to aortocaval compression and pregnancy-related vasodilation. These mechanisms contribute to the high incidence of PSH and underscore the importance of anticipating and preventing hypotension during cesarean delivery under spinal anesthesia [3,13].

Our hemodynamic findings are consistent with the literature evaluating the shock index family across acute care settings. SI and its derivatives reflect the relationship between HR and arterial pressure and have been associated with hemodynamic deterioration in hemorrhage, trauma, and sepsis. In obstetric populations, elevated prespinal SI has also been associated with an increased risk of hypotension after spinal anesthesia, although its discriminatory performance when used alone may be limited [2,4,5,6,14]. In the present study, SI and MSI showed the highest AUC values among the individual indices, whereas DSI had the highest sensitivity but relatively low specificity. These findings suggest that the indices may have different practical implications depending on whether sensitivity or specificity is prioritized.

The RJV-CI was also higher in patients who developed PSH. Ultrasound-derived jugular venous measurements may reflect variations in venous filling and have been investigated as noninvasive markers of volume status and fluid responsiveness. Previous studies in obstetric and nonobstetric populations have reported associations between greater internal jugular collapsibility and hypotension or fluid responsiveness [7,8,9,10,11,12,15]. In pregnant women, the IJV may offer a practical alternative to the IVC because it is superficial and can be assessed without interference from the gravid uterus. However, the predictive performance of jugular ultrasound parameters has varied across studies, likely because of differences in patient characteristics, respiratory conditions, patient positioning, and measurement techniques [7,12,16].

The models combining RJV-CI with SI or MSI had higher apparent and optimism-corrected AUC values than the corresponding individual predictors in this cohort. RJV-CI remained significantly associated with PSH after adjustment for the respective shock index in each model. These findings suggest that focused venous ultrasonography may provide additional information when interpreted together with routinely available hemodynamic indices. Nevertheless, the observed differences in discrimination were modest, and the findings should not be interpreted as establishing a clinical prediction rule.

Although the combined models showed numerically higher discrimination than the individual indices, the absolute increase in AUC was modest. Therefore, these findings should not be interpreted as evidence that RJV-CI-based models are ready to change routine perioperative management or replace current preventive strategies for postspinal hypotension. At present, the proposed measures may have a role in identifying patients for closer hemodynamic surveillance or consideration of individualized preventive measures; however, the observed thresholds should not be used as standalone clinical decision rules. Any future clinical application should be based on externally validated models that demonstrate added value beyond routinely available clinical information and standard guideline-based prophylactic strategies.

Future multicenter studies with larger and more diverse obstetric populations should externally validate the observed associations and evaluate whether the addition of RJV-CI to routinely available clinical variables provides clinically meaningful incremental value. Such validation should also determine whether the proposed measures improve risk assessment beyond maternal characteristics and baseline hemodynamic variables. Future studies should assess maternal symptoms, vasopressor requirements, and neonatal outcomes such as Apgar scores and umbilical cord blood gas measurements and should compare these measures with other bedside ultrasonographic or clinical risk assessment approaches. Prospective evaluation of whether risk-stratified preventive strategies improve maternal or neonatal outcomes would also be required before implementation in routine practice.

Limitations: Several limitations should be considered. First, this was a single-center study with a relatively small sample size and 36 PSH events. Although bootstrap internal validation was performed, model performance estimates may remain unstable, and external validation is required before the models and derived cutoff values can be considered for clinical use. In addition, the incidence of PSH and the derived cutoff values are conditional on the predefined composite definition of PSH used in this study. Alternative definitions or treatment targets for spinal hypotension may yield different event rates, operating thresholds, and model performance estimates. Second, the regression models included only RJV-CI and one shock index and therefore do not establish independence from other potentially relevant clinical predictors of PSH. Third, formal interobserver agreement for the ultrasound measurements was not assessed. Although measurements were obtained using a standardized protocol by trained investigators and averaged to reduce variability, RJV-CI may be influenced by probe pressure, patient positioning, respiratory pattern, and operator technique. Fourth, neonatal outcomes, including Apgar scores and umbilical cord blood gas values, were not collected as prespecified outcomes; therefore, the relationship between the identified maternal risk markers and neonatal well-being could not be evaluated. Finally, the modest numerical differences in discrimination between individual and combined models should be interpreted cautiously and do not support routine clinical implementation.

## 5. Conclusions

This prospective observational study found that prespinal bedside measures, including SI, MSI, DSI, and RJV-CI, were associated with the development of PSH in patients undergoing cesarean delivery under spinal anesthesia. In this cohort, models combining RJV-CI with SI or MSI showed numerically higher discrimination than the individual predictors, with this pattern retained after bootstrap internal validation.

The derived cutoff values of SI ≥ 0.74, MSI ≥ 0.98, and RJV-CI ≥ 30% were associated with PSH in the present cohort. However, these thresholds should be considered exploratory and should not be applied as routine clinical decision thresholds without external validation.

Overall, the findings suggest that RJV-CI may provide complementary information when interpreted alongside routinely available shock indices. Larger multicenter studies are needed to externally validate the observed associations, assess the reproducibility of the proposed cutoff values, and determine whether risk-based preventive strategies improve maternal or neonatal outcomes. Until such validation is available, these measures may be explored as adjunctive components of prespinal risk assessment rather than as standalone tools for directing clinical management.

## Figures and Tables

**Table 1:** Demographic and preoperative characteristics.

	PSH Group (*n* = 36)			Non-PSH Group (*n* = 40)			
Variable	Mean ± SD	Median (Min–Max)	95% CI	Mean ± SD	Median (Min–Max)	95% CI	*p* Value
Age (year)	28.17 ± 5.60	28.00 (18–40)	26.27–30.06	29.73 ± 5.69	30.00 (18–43)	27.90–31.55	0.234 *
Height (cm)	160.75 ± 6.56	160.00 (150–177)	158.53–162.97	161.40 ± 6.02	160.00 (145–175)	159.48–163.32	0.654 *
Weight (kg)	78.28 ± 13.72	79.50 (55–112)	73.64–82.92	79.53 ± 12.80	80.00 (57–110)	75.43–83.62	0.683 *
Body Mass Index (kg/m^2^)	30.37 ± 5.40	30.98 (18.38–41.14)	28.55–32.20	30.50 ± 4.37	31.42 (21.94–37.59)	29.10–31.89	0.913 *
ASA	2.03 ± 0.17	2.00 (2–3)	2.00–2.08	2.00 ± 0.00	2.00 (2–2)	2.00–2.00	0.304 **
Fasting duration (h)	10.44 ± 3.48	10.50 (3–16)	9.33–11.47	10.05 ± 3.73	10.00 (2–16)	8.85–11.22	0.722 **
Preoperative fluid administration (mL)	530.56 ± 523.62	500.00 (0–2000)	363.89–694.51	437.50 ± 479.68	0.00 (0–1000)	300.00–585.00	0.457 **
Hb (g/dL)	10.83 ± 1.17	10.90 (8.9–13.4)	10.44–11.22	11.10 ± 1.26	11.10 (8.3–13.1)	10.70–11.50	0.341 *
Htc (%)	34.17 ± 3.33	34.00 (29–41)	33.04–35.29	35.10 ± 3.40	35.00 (29–41)	34.01–36.19	0.231 *
MCV (fL)	83.69 ± 6.42	84.50 (68–95)	81.64–85.81	83.50 ± 8.21	84.00 (66–94)	80.75–85.90	0.811 **

* *t*-test, ** Mann–Whitney U test. CI: Confidence Interval, Hb: Hemoglobin, Htc: Hematocrit, MCV: Mean Corpuscular Volume, SD: Standard Deviation.

**Table 2:** Comparison of shock indices and right internal jugular vein collapsibility index.

	PSH Group (*n* = 36)			Non-PSH Group (*n* = 40)			
Variable	Mean ± SD	Median (Min–Max)	95% CI	Mean ± SD	Median (Min–Max)	95% CI	*p* Value
Shock Index	0.84 ± 0.14	0.81 (0.634–1.18)	0.79–0.89	0.71 ± 0.12	0.69 (0.51–1.0)	0.68–0.75	<0.001 **
Modified Shock Index	1.16 ± 0.21	1.14 (0.88–1.75)	1.10–1.24	0.99 ± 0.18	0.97 (0.71–1.47)	0.93–1.04	<0.001 **
Diastolic Shock Index	1.52 ± 0.28	1.47 (1.05–2.17)	1.44–1.62	1.32 ± 0.27	1.26 (0.84–1.90)	1.24–1.40	0.004 **
RJV-CI (%)	44.22 ± 18.54	38.88 (11.25–75.26)	37.93–50.02	31.18 ± 15.74	28.40 (8.03–78.43)	26.80–36.63	0.002 **
RJVmax/CA	1.48 ± 0.44	1.40 (0.80–2.62)	1.33–1.63	1.43 ± 0.49	1.48 (0.12–2.33)	1.28–1.59	0.657 *
RJVmin/CA	0.84 ± 0.40	0.80 (0.26–1.75)	0.70–0.97	1.01 ± 0.49	0.95 (0.07–2.0)	0.85–1.17	0.091 *
RJVmean/CA	1.16 ± 0.38	1.10 (0.54–1.95)	1.03–1.29	1.22 ± 0.48	1.23 (0.09–2.16)	1.07–1.38	0.520 *

* *t*-test, ** Mann–Whitney U test. CA: Carotid Artery, CI: Confidence Interval, RJV: Right internal Jugular Vein, RJV-CI: Right internal Jugular Vein Collapsibility Index, SD: Standard Deviation.

**Table 3:** Univariable ROC analysis.

Variable	AUC	95% CI	Optimal Cutoff	Sensitivity	Specificity
Shock Index	0.757	0.643–0.859	0.742	0.750	0.650
Modified Shock Index	0.747	0.628–0.851	0.976	0.861	0.525
Diastolic Shock Index	0.693	0.563–0.808	1.254	0.917	0.475
RJV-CI	0.709	0.584–0.821	30.488	0.778	0.575

AUC: Area under the curve, CI: Confidence Interval, RJV-CI: Right internal Jugular Vein Collapsibility Index.

**Table 4:** Multivariable logistic regression models and performance metrics.

Model and Variables	Odds Ratio (95% CI)	*p* Value	AUC (95% CI)	Optimism-Corrected AUC †	Calibration (H-L Test) ‡
Model A					
RJV-CI ^a^	1.034 (1.003–1.067)	0.034 *	0.790 (0.688–0.886)	0.775	*p* = 0.559
Shock Index ^b^	1.96 (1.25–3.06)	0.003 *			
Model B					
RJV-CI ^a^	1.037 (1.005–1.069)	0.021 *	0.787 (0.681–0.890)	0.774	*p* = 0.597
Modified Shock Index ^b^	1.54 (1.14–2.09)	0.005 *			
Model C					
RJV-CI ^a^	1.041 (1.010–1.073)	0.010 *	0.763 (0.677–0.884)	0.748	*p* = 0.398
Diastolic Shock Index ^b^	1.29 (1.06–1.57)	0.010 *			

AUC: Area under the curve; CI: Confidence Interval; RJV-CI: Right internal jugular vein collapsibility index. * Statistically significant (*p* < 0.05). ^a^: Odds ratios for RJV-CI corresponding to a 1% increase. ^b^: Odds ratios for shock indices corresponding to a 0.1 unit increase. †: The optimism-corrected AUC was estimated via bootstrap internal validation (1000 resamples) to account for potential overfitting. ‡: Hosmer–Lemeshow goodness-of-fit test; *p* > 0.05 indicates no evidence of lack of fit.

## Data Availability

The data presented in this study are available upon request from the corresponding author. The data are not publicly available because they contain individual-level clinical information and are subject to ethical and privacy restrictions.

## Supplementary Materials

The following supporting information can be downloaded at: https://www.mdpi.com/article/10.3390/jcm15155842/s1, Supplementary Figure S1. Exploratory decision-curve analysis for individual and combined prespinal models. Net benefit is shown across a range of threshold probabilities for SI alone, MSI alone, DSI alone, RJV-CI alone, Model A (RJV-CI + SI), Model B (RJV-CI + MSI), Model C (RJV-CI + DSI), and the treat-all and treat-none strategies. Because this analysis was performed in the development cohort, the findings should be interpreted as exploratory. Supplementary Figure S2. Exploratory apparent calibration plots for the combined prespinal models. Observed proportions of post-spinal hypotension are plotted against predicted probabilities for Model A (RJV-CI + SI), Model B (RJV-CI + MSI), and Model C (RJV-CI + DSI). The dashed diagonal line represents ideal calibration. Because these plots were generated in the development cohort, they should be interpreted as exploratory.

## Abbreviations

The following abbreviations are used in this manuscript:

AUC	Area under the curve
CA	Carotid artery
CI	Confidence interval
DAP	Diastolic arterial pressure
DSI	Diastolic shock index
EPV	Events per variable
HR	Heart rate
IJV	Internal jugular vein
IJV-CI	Internal jugular vein collapsibility index
IVC	Inferior vena cava
MAP	Mean arterial pressure
MSI	Modified shock index
NIBP	Noninvasive blood pressure
OR	Odds ratio
PSH	Postspinal hypotension
RJV	Right internal jugular vein
RJV-CI	Right internal jugular vein collapsibility index
RJVmax	Maximum right internal jugular vein diameter
RJVmean	Mean right internal jugular vein diameter
RJVmin	Minimum right internal jugular vein diameter
ROC	Receiver operating characteristic
SAP	Systolic arterial pressure
SD	Standard deviation
SI	Shock index
