# Supplementary material for: Association of Pre-Spinal Shock Indices and Right Internal Jugular Vein Collapsibility Index with Post-Spinal Hypotension During Cesarean Delivery: A Prospective Observational Study
Source: J Clin Med. 2026 Jul 26;15(15):5842. doi: 10.3390/jcm15155842 (PMC13467304; doi:10.3390/jcm15155842)
Supplement: Supplementary file 1 [file jcm-15-05842-s001.zip › jcm-4424327-supplementary.pdf]

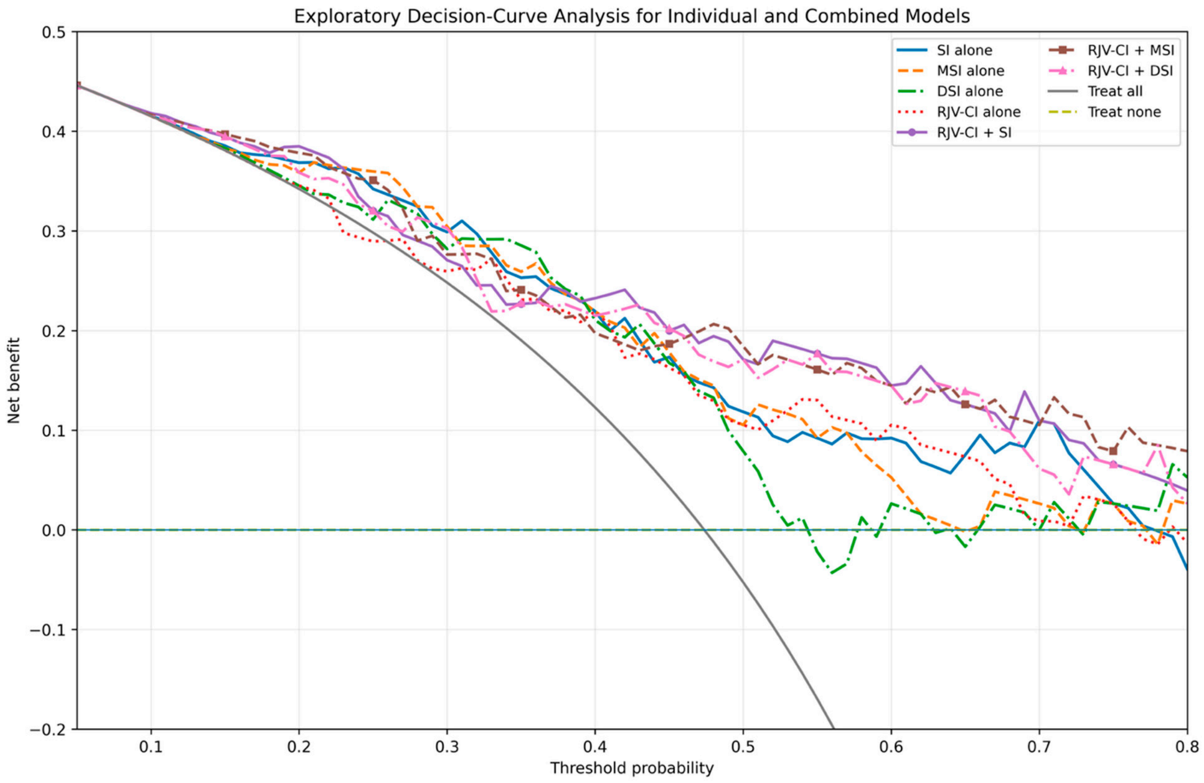

Supplementary Figure S1. Exploratory decision-curve analysis for individual and combined prespinal models. Net benefit is shown across a range of threshold probabilities for SI alone, MSI alone, DSI alone, RJV-CI alone, Model A (RJV-CI + SI), Model B (RJV-CI + MSI), Model C (RJV-CI + DSI), and the treat-all and treat-none strategies. Because this analysis was performed in the development cohort, the findings should be interpreted as exploratory.

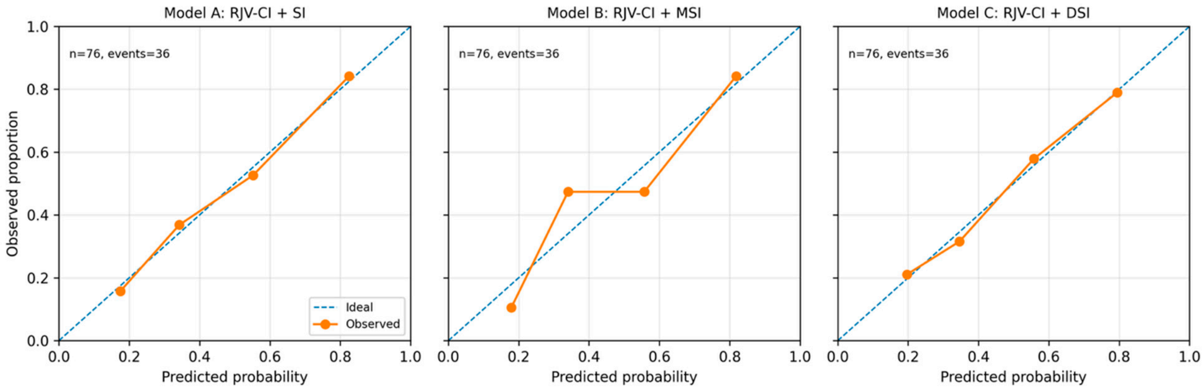

Supplementary Figure S2. Exploratory apparent calibration plots for the combined prespinal models. Observed proportions of post-spinal hypotension are plotted against predicted probabilities for Model A (RJV-CI + SI), Model B (RJV-CI + MSI), and Model C (RJV-CI + DSI). The dashed diagonal line represents ideal calibration. Because these plots were generated in the development cohort, they should be interpreted as exploratory.
